# New Mode of Treating Dyspepsia

**Published:** 1874-01

**Authors:** 


### A New Mode of Treating Dyspepsia.

The first number of *The Archives of Scientific and Practical Medicine*, the new monthly edited by Dr. Brown-Sequard, and published by the Lippincotts, contains, among other able and interesting articles, one in which the editor describes a novel mode of treatment which he first tried with perfect success in a very bad case of dyspepsia in 1851, and which has since been tested, with more or less satisfactory results, in many cases of dyspepsia, chlorosis, and anæmia. The following is an extract from the account of the first case :

" After a few days, finding that he had not improved, I decided to try a radical change of his alimentation, as regards the quantity of food to be taken at a time. Instead of *three* meals a day, I made him take *sixty* or more. Every twelve or fifteen minutes he took two or three mouthfuls of solid food, chiefly meat and bread. He drank a little less than a wineglass of Bordeaux wine and water every thirty or forty minutes. On the very first day this mode of alimentation was begun his digestive troubles disappeared, and within a week he was so well that he returned to Paris. . . . He continued the same mode of alimentation for almost three weeks, and then gradually diminished the number of his homeopathic meals, and increased the amount taken at each of them, until in about eight or ten days he came to eat only three times a day, and a full meal at each time."

The following paragraphs will serve to give the reader a clearer idea of the treatment commended :

" The plan consists in giving but very little of solid or fluid food or any kind of drink at a time, and giving these things at regular intervals of from ten to twenty or thirty minutes. All sorts of food may be taken in that way, but during the short period when such a trial is made, it is obvious that the fancies of the patients are to be laid aside, and that nourishing food, such as roasted or broiled meat, and especially beef, mutton, eggs, well-baked bread, and milk, with butter and cheese, and a very moderate quantity of vegetables and fruit, ought to constitute the dietary of the patients we try to relieve. This plan should be pursued two or three weeks, after which the patient should gradually return to the ordinary system of eating three times a day. . . .

The most varied diet as regards the kind of food can be followed under this plan as well as when one has only two or three meals a day. The only absolutely essential points are, that the amount of food taken every 10, 15, 20 or 30 minutes be very small (from one to four mouthfuls), and that the quantity of solid food in a day be from 32 to 40 ounces, or a little less when, instead of water, the patient drinks beef-tea or milk."

Dr. Brown-Sequard considers that the facts observed under this treatment confirm "the view that we are naturally organized, like most, if not all animals, to eat very frequently, and not, as we do, two, three or four times a day ;" and that " functional dyspepsia, when once it has begun (never mind by what cause), is kept up and increased by distention of the walls of the stomach." It might be supposed that there would be trouble from the distention of the stomach on the return to the ordinary system of meals, after several weeks of the treatment described, but in no case has he found this to occur.

